# Effect of Secondary Cold Reduction Rates on Microstructure, Texture and Earing Behavior of Double Reduction Tinplate

**DOI:** 10.3390/ma14144040

**Published:** 2021-07-20

**Authors:** Peng Tian, Luhai Liao, Guoming Zhu, Yonglin Kang

**Affiliations:** School of Material Science and Engineering, University of Science and Technology Beijing, Beijing 100083, China; tpchbr@163.com (P.T.); liaoluhai2021@163.com (L.L.)

**Keywords:** secondary cold reduction, texture characterization, earing behavior, double reduction tinplate

## Abstract

In order to evaluate the effect of secondary cold reduction rate on the drawing performance of double reduction tinplate and explain the mechanism, a detailed investigation into the microstructural characterization, dissolved carbon atoms, texture characterization by an X-ray powder diffractometer (XRD) and electron backscatter diffraction (EBSD), and earing behavior were carried out with different secondary cold reduction rates of 15%, 20% and 25% for double reduction tinplate. The experimental results indicate that 15% secondary cold reduction rate could obtain a better drawing performance because there are no holes and cracks at the microstructure, and the content of dissolved carbon atom is relatively low; at the same time, it has a better texture distribution and low earing coefficient.

## 1. Introduction

With the competition of the packaging market and the progress of secondary cold rolling technology, double reduction (DR) tinplate, as a new generation of green metal packaging materials, has become a new research hotspot with its thinner thickness, higher strength, surface precision and better stamping performance [1]. Secondary cold rolling technology is a kind of ultrathin strip production process, where a hot-rolled strip is further thinned by 15–40% secondary reduction rate after pickling, first cold rolling and annealing. It can further reduce the thickness of tinplate from 0.24 mm to 0.14 mm [2], and achieve the goal of reducing the cost and weight of packaging materials. Relevant research [3,4] shows that each process of DR tinplate production plays a large or small role in the microstructure, mechanical properties, texture and earing behavior of annealed plate or tinplate, including the chemical composition of C, B, Ti, N [5,6], the hot rolling process [7,8,9], first cold reduction and the continuous annealing process [10], first cold reduction and the batch annealing process [11], secondary cold reduction [12], etc. Due to the excessive earing of DR tinplate after drawing or deep drawing, the trimming process is needed and results in the waste of raw materials. In order to improve the yield and lower the production cost, it is required to develop “non-earing and ultra-low earing” tinplate materials [13].

The effects of first cold rolling and secondary cold rolling on the mechanical properties and formability of DR tinplate are completely different. The raw material used for first cold rolling is hot-rolled plate, and the performance optimization of DR tinplate is indirectly realized by producing a cold-rolled texture to affect the texture of annealed plate. However, the raw material used for secondary cold rolling is annealed plate, and the new cold rolling texture is produced on the basis of annealed texture to ensure the mechanical properties and formability of DR tinplate. Earing propensity of a material can be determined by drawing a cup or may be predicted from the crystallographic texture or from the planar anisotropy value (Δr) determined from tension tests [14]. The annealed plate is thicker after first cold rolling and its elongation is over 30%, so the thickness anisotropy value of different directions (r_0_, r_45_, r_90_), the average plastic strain ratio (r_m_) and Δr or earing test are used to evaluate the drawing performance. While the thickness of DR tinplate is thinner and the elongation is less than 5% after secondary cold rolling, the drawing performance can only be reflected by the semi-quantitative analysis of the plastic strain ratio of each texture or earing test. Therefore, the present study was undertaken to explore the microstructure characteristics, solid solution carbon content, texture characteristics and earing behavior of DR tinplate with different secondary cold reduction rates, and to explain their relationship, so as to provide technical support for the development of tinplate with low earing and high quality.

## 2. Materials and Methods

For this study, three samples of commercially produced DR tinplate (DR8) were obtained. The samples were from three heats of continuously cast low-carbon aluminum-killed steel and their chemical composition is given in Table 1.

The processing parameters for the three steels are provided with Table 2, in which the finisher delivery temperature (FDT), coiling temperature (CT), thickness of the hot-rolled strip, first cold reduction rate, continuous annealing temperature (CAT), thickness of the annealed strip, secondary cold reduction rate, and thickness of double reduction tinplate are denoted by FDT, CT, t_0_, ε_1_, CAT, t_1_, ε_2_, and t_2_, respectively. The same hot rolling, cold rolling and continuous annealing processes were experienced before the secondary cold roll, and then these annealed sheets were rolled into double reduction tinplate with a different thickness at ε_2_ of 15%, 20% and 25% on a double stand secondary cold mill.

The specimens were cut from secondary cold reduction tinplate under different reduction rates. The samples’ surfaces along the rolling direction were mounted, polished, and then etched in 4 vol% nitric solution to observe the microstructure under scanning electron microscopy (SEM) (Quanta FEG450, FEI corporation, Hillsboro, OR, USA). The content of dissolved carbon in the different steels was measured by an X-ray photo electron spectrometer (XPS) (Axis Ultra DLD, Kratos, Manchester, UK), to eliminate the influence of surface contaminants and ensure the test results were close to the internal components of the samples; polished specimens were cleaned by an ultrasonic cleaner (KH50B, Hechuang, Jiangsu, China) for 10 min and etched with argon ions for 40 s before testing. Then, curve fitting was used with XPS Peak 4.1 and the Shirley background was used for the curve deconvolution. The binding energy of C 1s in the spectrum was determined based on the NIST database. The macroscopic texture distribution of the samples was determined by an X-ray powder diffractometer (XRD) (MXP21VAHF, Mac, Tokyo, Japan). The preparation for electron backscatter diffraction (EBSD) samples includes normal mechanical polishing and the electrolytic polishing with the solution containing 5 vol. % perchloric acid and 95 vol. % acetic acid. Earing tests were carried out on a universal sheet-testing machine (BCS-30, Beihang, Beijing, China). The preparation of samples, operation of the earing test and treatment of test results are in accordance with the national standard GB/T 15825.7-2008.

## 3. Results

### 3.1. Microstructural Characterization

**Figure 1:**
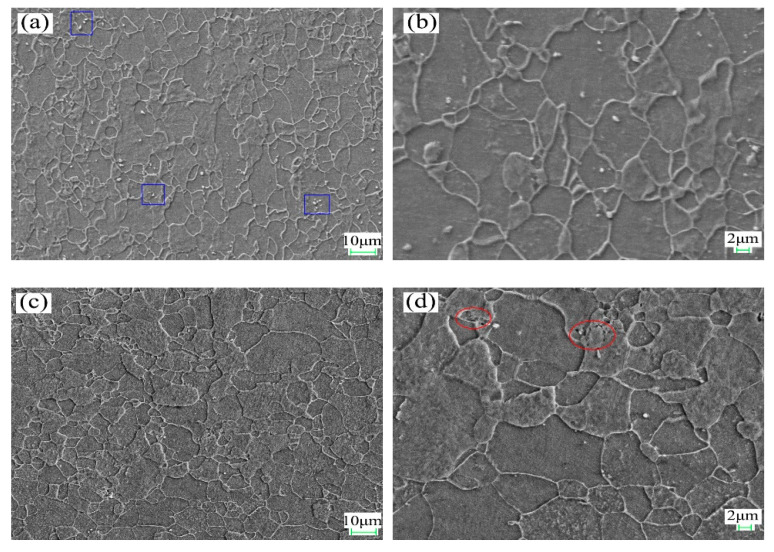

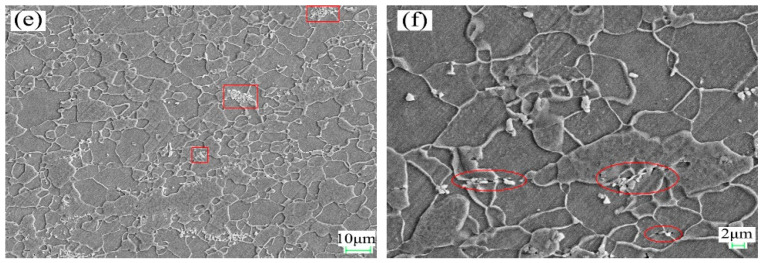
SEM images with different secondary cold reduction rates: (**a**) 15%, 2000×; (**b**) 15%, 5000×; (**c**) 20%, 2000×; (**d**) 20%, 5000×; (**e**) 25%, 2000×; (**f**) 25%, 5000×.

The microstructure of DR tinplate with different secondary cold reduction rates of 15%, 20% and 25% is shown in Figure 1. The average ferrite grain sizes with reduction rates of 15%, 20% and 25% was 7.4 μm, 7.0 μm and 6.8 μm, respectively, and the aspect ratio of ferrite grain was 1.68, 1.75, and 1.77, respectively. The results show that the grain size and aspect ratio of the three samples varied slightly. With the increase in the secondary cold reduction rate, the morphology of ferrite grains evolved from an annealed equiaxed shape to an elongated shape along the rolling direction. A small amount of cementite precipitated in the form of cluster (see blue squares in Figure 1a) or particle (see Figure 1b) in ferrite for steel A, the size of spherical cementite was about 0.4 μm. There was little cementite precipitation in ferrite for steel B, but holes appeared around some cementite, as shown in the red ovals in Figure 1d. Parts of cementite precipitated in the form of lamellar (see red squares in Figure 1e), cluster (see blue ovals in Figure 1f) or spherule at the ferrite grain boundary for steel C, and the size of spherical cementite was about 1 μm. The occurrence of lamellar pearlite was mainly related to the relatively high carbon content of steel C. At the same time, micro cracks along the rolling direction preferentially appeared in the cluster cementite, as shown in the red ovals in Figure 1f. Compared with steel A and steel B, it could be concluded that with the increase in the secondary cold reduction rate, due to dislocations piled up at the boundaries resulting in stress concentration, holes appeared preferentially at the phase interface [15,16], and then developed into cracks, which would lead to the decline of formability. From a comparative analysis of steel A, steel B and steel C, it was supposed that the size and spacing of cementite particles were the main factors that deteriorated the formability of DR tinplate besides the increase in the secondary reduction rate.

As for the effect of cementite on the formability of the material, it was supposed that changes in the size and spacing of cementite particles in a hot band would cause changes in the number, the distribution and the individual size of the “constrained deformation regions” in the cold-rolled sheet [17]. This is as the large grain cementite or lamellar pearlite precipitated at the grain boundary more easily becomes the source of micro cracks and would lead to the non-uniformity deformation during the cold rolling process. It could be concluded that the formability of DR tinplate deteriorated with the increase in the secondary cold reduction rate and the change of cementite morphology.

### 3.2. Analysis of the Dissolved Carbon Atoms

**Figure 2:**
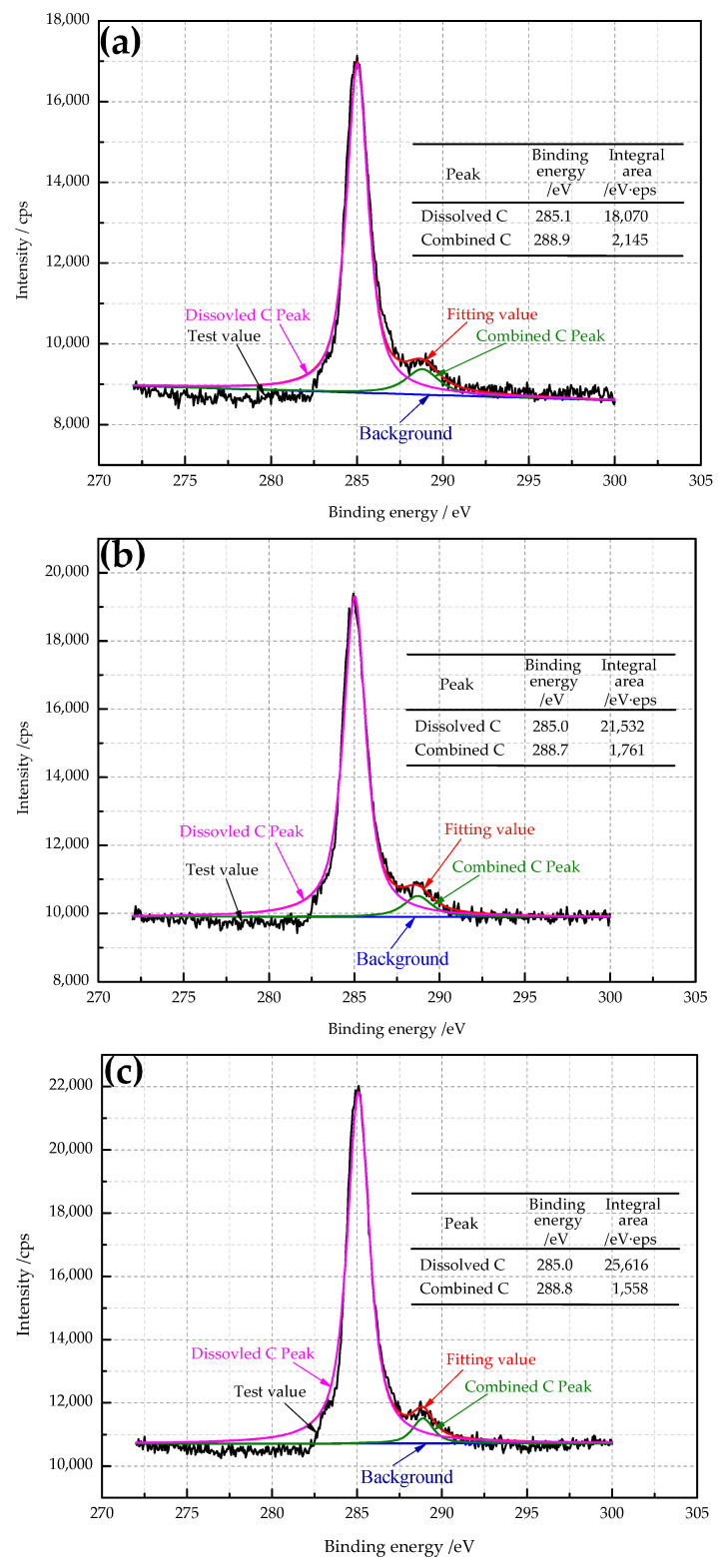
The spectra of carbon 1s by XPS and its fitting curves with different secondary cold reduction rates: (**a**) 15%; (**b**) 20%; (**c**) 25%.

The spectra of carbon 1s by XPS and its fitting curves are exhibited in Figure 2. According to the NIST data [18], 284.8 eV and 288.2 eV are the binding energies of dissolved carbon atoms and compound carbon atoms, respectively. For this experiment, the binding energies of dissolved carbon atoms and compound carbon atoms were about 285.0 eV and 288.8 eV. The difference between the test results and the standard values lay in the subtle differences in the test environment and materials. It can be seen from Figure 2 that the C 1s peak spectra with different secondary cold reduction rates have similar peak shapes: the peak intensity of dissolved carbon atoms from low to high was A < B < C. In order to better compare the relative content of dissolved carbon atoms, the integral area of dissolved carbon peak was used for comparison; the integral area from low to high was A < B < C, which indirectly indicated that the content of dissolved carbon atoms in DR tinplate from low to high was A < B < C, but the content of compound carbon atom shows the opposite rule.

### 3.3. Texture Analysis by XRD

**Figure 3:**
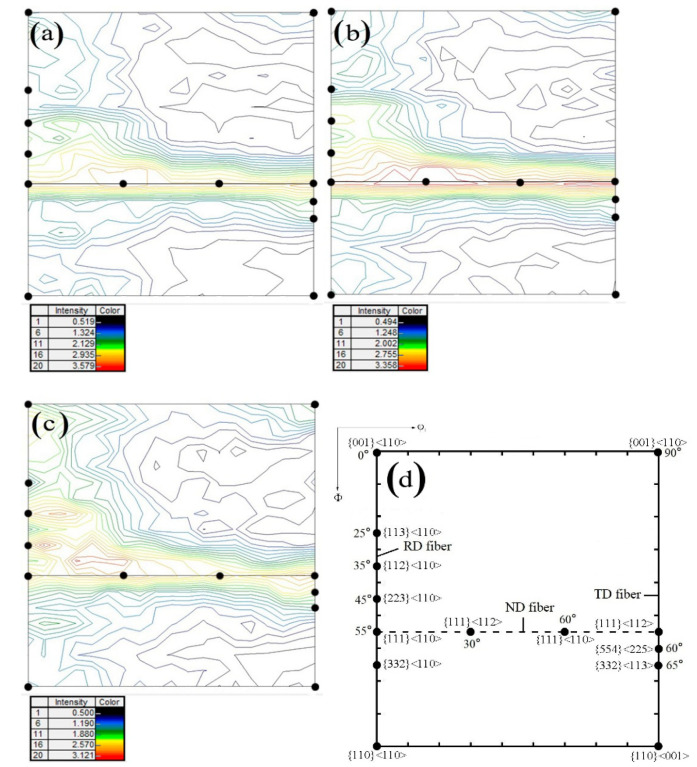
ODF sections (φ2 = 45°) with different secondary cold reduction rates: (**a**) 15%, (**b**) 20%, (**c**) 25%, (**d**) the important orientation positions.

Orientation distribution function (ODF) section is the most representative section to express the texture of cubic metal materials, which can reflect a series of important orientation positions. Figure 3 shows the φ2 = 45° sections of the ODF derived from XRD for DR tinplate with different secondary cold reduction rates. The textures of all samples were mainly composed of incomplete α fiber texture parallel to the rolling direction (RD), complete γ fiber texture along the normal direction (ND) and partial fiber texture belonging to the transverse direction (TD); the difference in color was the higher ND texture density for steel B and two higher density regions along the RD for steel C.

**Figure 4:**
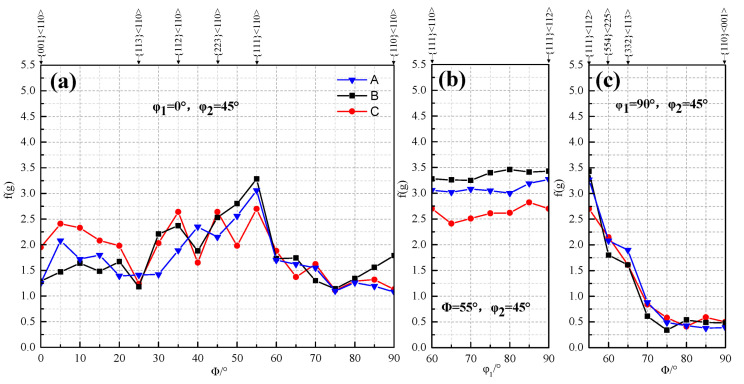
Texture distribution with different secondary cold reduction rates on the different oriented lines: (**a**) RD; (**b**) ND; (**c**) TD.

The texture distribution with different secondary cold reduction rates on the different oriented lines is exhibited in Figure 4. The difference in the RD orientation line was that the strongest point of texture for steel A and B was {111} <110>, but for steel C, they were three high extremum points, which are {112} <110>, {223} <110> and {111} <110>, respectively. At the same time, the intensity function value of near {001} <110> texture was also higher, and the value of {112} <110> texture increased with the increase in the secondary cold reduction rates [19]. The higher intensity RD fiber texture, especially {001} <110>, {112} <110> not only reduced the r_m_ value of the material, but also promoted the earing direction of 45° [3]. On the ND orientation line, the intensity function value of {111} <112> for steel A and B was greater than that of {111} <110>, which made it easy to produce ear on the rolling direction of 0°/60°. However, the {111} texture for steel C was difficult to produce ear on the rolling direction of 0°/60° because {111} <112> was continuously transformed into {111} <110> with the increase in the secondary cold reduction rate. On the TD orientation line, the texture distribution of different secondary cold reduction rates was similar, and the intensity function value decreased rapidly with the increase in ϕ. However, both {554} <225> and {332} <113> had certain strength function values, which would produce ear on the rolling direction of 0°/60° and 30°/90°, respectively. This could balance ear on the rolling direction of 45° and reduce the earing coefficient.

### 3.4. Texture Analysis by EBSD

**Figure 5:**
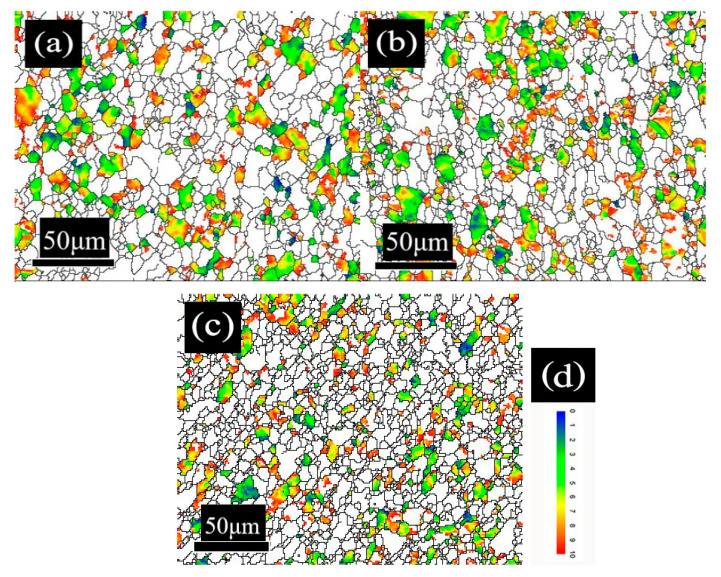
Misorientation distribution of desired {111} grains with different secondary cold reduction rates: (**a**) 15%; (**b**) 20%; (**c**) 25%; (**d**) color legend.

To further illustrate the proportion of the favorable textures, the texture component bar of Channel 5 was used to separate the grains of {111} and near {111} orientations (including {554} <225> and {332} <113>) with different colors. The misorientation distribution of desired {111} grains with different secondary cold reduction rates is exhibited in Figure 5. The white acted as a background. The closer the color was to red, the larger the grain misorientation was; the maximum misorientation is 15°. The volume fraction of these {111} and near-{111} textures with different secondary cold reduction rates of 15%, 20% and 25% was 26.3%, 27.3% and 22.1%, respectively. With the increase in the secondary cold reduction rate, the volume fraction of these {111} and near—{111} textures show a trend of rising first and then decreasing significantly. The results of quantitative analysis are consistent with those of qualitative analysis in Figure 3 and Figure 4b. In addition, it can be seen from Figure 5 that the number of grains increased with the increase in the secondary cold reduction rate, but the grain sizes decreased, which was consistent with Figure 1.

**Figure 6:**
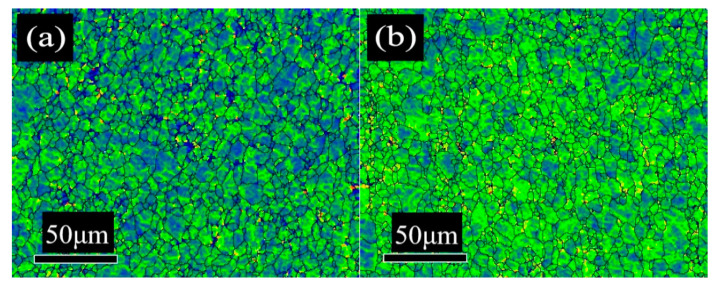

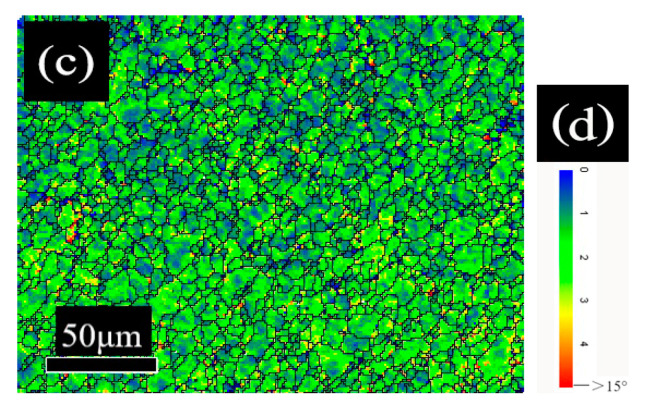
Local misorientation distribution with different secondary cold reduction rates: (**a**) 15%; (**b**) 20%; (**c**) 25%; (**d**) color legend.

The local misorientation can be used to analyze the misorientation change in the grain of the plastic deformation metal, which is related to the dislocation density of the material, so it can indirectly reflect the relative strength of the residual stress [13]; that is, it can indirectly reflect the change of the secondary cold reduction rate. Figure 6 shows the local misorientation distribution with different secondary cold reduction rates, the closer the color was to red, the larger the grain misorientation was; the maximum misorientation is 15°. The blue region decreased with the increase in the secondary cold reduction rate. When the secondary cold reduction rate reached 25%, the color of some grains reached red, as shown in Figure 6c, implying an increase in the substructure or the dislocation density.

**Figure 7:**
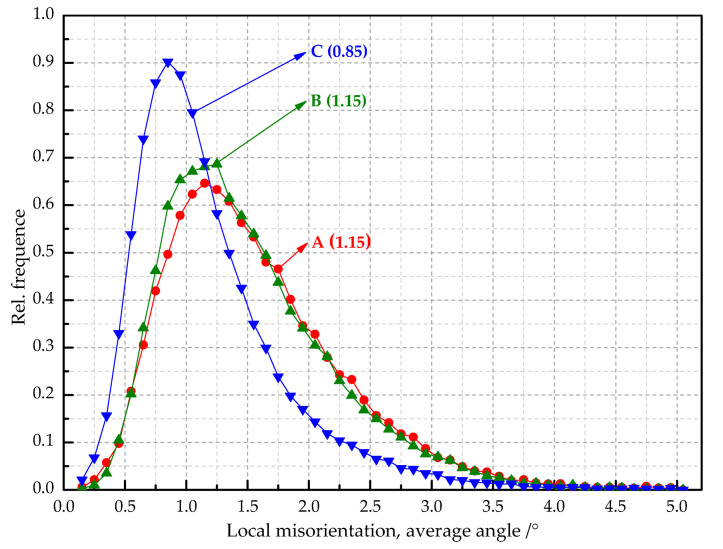
Distribution curves of local misorientation with different secondary cold reduction rates.

In order to show the difference in local misorientation, the distribution curves of local misorientation with different secondary cold reduction rates were shown in Figure 7. The distribution curves were of unimodal distribution, and the values in the brackets represented the mean value of the local misorientation. With the increase in the secondary cold reduction rate, the peak value of local misorientation decreased, but the mean value increased. The mean values of steel B and steel C are similar: both are 1.15, because local holes and cracks appear in the microstructure (see Figure 1); part of the internal residual stress was released, so the change was very small.

### 3.5. Earing Behaviors

**Figure 8:**
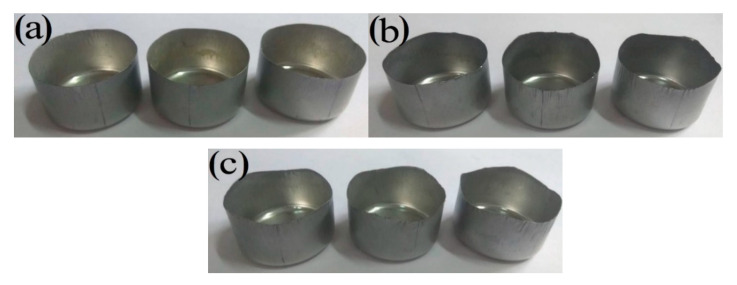
Experimentally drawn cups with different secondary cold reduction rates: (**a**) 15%; (**b**) 20%; (**c**) 25%.

Figure 8 shows the experimentally drawn cups with different secondary cold reduction rates after the round pieces were punched. It is shown that the sample with a secondary cold reduction rate of 15% had small ears, and that of 20% and 25% had obvious ears with a certain angle to the rolling direction.

**Figure 9:**
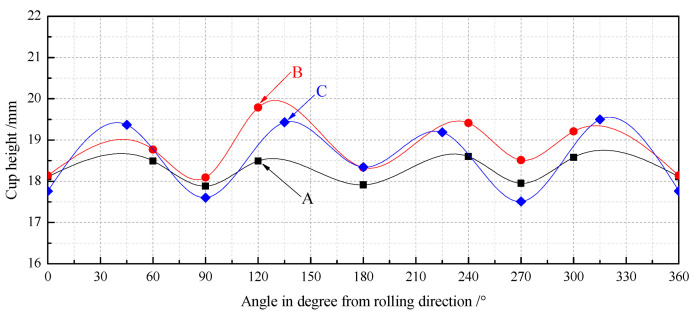
Earing heights at varied angels from rolling direction.

In order to show the change of the direction and height of the ears more intuitively, the earing heights of varied angels from rolling direction are exhibited in Figure 9. Several important indexes to characterize the earing propensity are clearly presented in Table 3 in which $\bar{h}$t, $\bar{h}$v, $\bar{h}$e, Δhmax, and Ze devoted the average height of ear peak, ear valley, earing, maximum ear height, and earing coefficient, respectively. According to Figure 9 and Table 3, the earing direction of steel A was 0°/60°, its $\bar{h}$e and Δhmax were less than 1 mm, and Ze was no more than 5%, so 15% reduction rate of secondary cold was considered the best process for DR tinplate. These predictions were in general agreement with experimental observations [20]. The earing direction of steel C was 45° and its earing propensity was the worst, because it not only had the highest $\bar{h}$t, $\bar{h}$e and Δhmax, but also has the lowest $\bar{h}$v, so its Ze was the highest. Steel B was between steel A and steel C, which was in the transition stage. The earing propensity from good to bad was: steel A > steel B > steel C. That is to say, with the increase in the secondary cold reduction rate, $\bar{h}$t, $\bar{h}$e, Δhmax and Ze increased significantly, while the earing propensity decreased significantly, and the earing direction gradually changed from 0°/60 to 45°.

## 4. Discussion

The most important factor that affects the earing number, height and direction is the distribution of texture in steel sheet. Each texture has a different influence on the drawing performance. As a deep drawing or ultra-deep drawing sheet, we need as high an r_m_ value as possible and Δr to be as low as possible, because the lower r_m_ value will lead to the faster occurrence of punching crack and the higher |Δr| will produce a large ear. In order to reduce the ear height after stamping, improve the product yield rate, and obtain low ear tinplate, it is necessary to explore the best texture distribution of sheet. Table 4 shows the r_m_, Δr and earing direction for main texture components [3,21]. According to Table 4, {111} <110>, {111} <112>, {554} <225>, {223} <110> and {332} <113> contribute the most to r_m_, and the decrease in formability caused by a {001} texture can only be compensated by 4-5 times of {111} texture. Therefore, reducing {001} texture will greatly improve the drawing performance. At the same time, texture balances between (111) [112] and (111) [110] orientations, and those between (554) [225] and (211) [011] orientations were found to be quite effective in eliminating earing [22].

In the cold rolling process, the effect of dissolved carbon on the texture of DR tinplate was mainly shown in the following aspects:

- The dissolved carbon and nitrogen atoms would bind dislocation and hinder the rotation and sliding of grains, thus preventing the development of {111} texture [23]. When the steel with a high content of dissolved carbon atoms was cold rolled, the strongest point of texture on RD orientation line tended to appear at {112} <110>~{223} <110>, rather than at the favorable {111} <110> texture;
- The dissolved carbon and nitrogen atoms would result in dynamic strain aging, which not only retained the {001} <110> texture components, but also increased the nucleation rate of the grains with unfavorable orientation [24]. Combined with Figure 2, steel C has the highest dissolved carbon content, the lowest {111} texture strength, and the highest {001} <110> texture strength, which is also in good agreement with the experimental results of reference [5,25]. This was because the content of Mn in steel C was also high; carbon, nitrogen and manganese were easy to form C-Mn or N-Mn-dissolved displacement atomic pairs, which greatly reduced the annealing {111} texture density of the steel sheet. According to Figure 2 and Table 3, with the increase in dissolution carbon content, the earing propensity gradually decreased.

In conclusion, the excessive secondary cold reduction rate was unfavorable to reduce the earing propensity, because the density of the RD texture would increase with the increase in the reduction rate and the {111} <112> texture would continue to change into the {111} <110> texture, resulting in the change of earing direction. To the contrary, appropriate secondary cold reduction rate would not only improve the strength of the plate, but also kept the earing coefficient at a low level. The reasons why the 15% reduction rate had good drawing performance were that it had:

- The minimum RD texture density, the smaller {111} <110> texture density and the minimum {223} <110> texture density;
- The minimum ND texture density, the equal density of {111} <110> texture and {111} <112> texture;
- The {554} <225> texture with certain density. The most ideal texture distribution which was most useful for reducing earing coefficient was not necessarily to obtain a higher {111} texture intensity, but to obtain a higher {111} texture density on the premise of minimizing RD texture.

The reason for the poor drawing performance of the 25% reduction rate was that the {223} <110> texture density is higher than that of {554} <225>, and {554} <225> texture could not make up for the adverse effect of {223} <110> texture.

The earing direction at 45° after being stamped showed that the RD texture played a leading role; it can be seen from Figure 4 that the density of the RD texture of steel C was the highest, and the density of ND texture was the lowest. It could be concluded that the decrease in non-{111} texture and the increase in {111} fiber texture are in favor of the decrease in earing coefficient, accompanied with the change of the earing position from 0°/60° to 45°.

## 5. Conclusions

DR tinplate with different secondary cold reduction rates of 15%, 20% and 25% was studied, and a detailed investigation of the microstructural characterization, dissolved carbon atoms, texture characterization by XRD and EBSD, and earing behavior was carried out. The following conclusions were drawn:

- Microstructure observations of the samples showed that, with the increase in the secondary cold reduction rate and the change of the size and spacing of cementite particles, due to dislocations piled up at the interface of ferrite and cementite resulting in stress concentration, holes appeared preferentially at some weak phase interfaces, and then developed into cracks, so that the formability of the material decreased.
- The existence of dissolved carbon atoms hinders the rotation of grains during the cold rolling process, and the increase in dissolved carbon content weakens the strength of the {111} texture component and enhance the strength of {001} <110> texture component, which will eventually lead to the decrease in earing propensity.
- With the increase in the secondary cold reduction rate, the volume fraction of these {111} and near-{111} textures show a trend of rising first and then decreasing significantly. The higher the secondary cold reduction rate, the more local misorientation increases, the peak value decreases and the mean value increases.
- With the increase in the secondary cold reduction rate, the average height of ear peak, earing, maximum ear height, and earing coefficient increased significantly; meanwhile, the earing direction changed from 0°/60° to 45°, and the earing propensity decreased significantly. Comprehensive consideration showed that 15% of secondary cold reduction rate could obtain a better drawing performance.

## Figures and Tables

**Table 1:** Chemical composition of investigated materials (mass fraction, %).

Steel	C	Si	Mn	P	S	Al	N	Fe
DR8	0.04–0.08	≤0.03	0.2–0.4	≤0.020	≤0.010	0.03–0.06	≤0.008	balance
A	0.045	0.017	0.26	0.013	0.006	0.044	0.0046	balance
B	0.042	0.010	0.22	0.011	0.007	0.050	0.0055	balance
C	0.063	0.011	0.31	0.015	0.001	0.040	0.0053	balance

**Table 2:** Parameter control of production process.

Steel	FDT (°C)	CT (°C)	t_0_ (mm)	ε_1_ (%)	CAT (°C)	t_1_ (mm)	ε_2_ (%)	t_2_ (mm)
A	880	680	2	90	680	0.2	15	0.17
B	880	680	2	90	680	0.2	20	0.16
C	880	680	2	90	680	0.2	25	0.15

**Table 3:** Several important indexes to characterize the earing propensity.

**Steel**	$\bar{h}$t (mm)	$\bar{h}$v (mm)	$\bar{h}$e (mm)	hmax (mm)	Ze (%)	Earing Direction (°)
A	18.54	17.96	0.58	0.75	3.2	0/60
B	19.30	18.26	1.03	1.48	5.6	0/60
C	19.37	17.80	1.57	1.97	8.8	45

**Table 4:** The r_m_, Δr and earing direction for main texture components.

Texture Component	r_m_	Δr	Earing Direction (°)
{001}<110>	0.4	−0.8	45
{114}<110>	1.2	−2.1	45
{113}<110>	1.0	−1.7	45
{112}<110>	2.1	−2.7	45
{223}<110>	2.5	−2.0	45
{111}<110>	2.6	0.0	30/90
{111}<112>	2.6	0.0	0/60
{554}<225>	2.6	1.1	0/60
{332}<113>	2.7	1.9	0/60

## Data Availability

The data presented in this study are available on request from the corresponding author.

## References

[1] Dey S., Agrawal M.K. (2016). Tinplate as a sustainable packaging material: Recent innovation and developments to remain environment friendly and cost effective. Int. J. Res. IT Manag. Eng..

[2] Čižmár I.M. (2015). Analysis of mechanical properties and their comparison against required parameters when producing DR tinplate sheet. Transf. Inovácií.

[3] Ray R.K., Jonas J.J., Hook R.E. (1994). Cold rolling and annealing textures in low carbon and extra low carbon steels. Int. Mater. Rev..

[4] Hutchinson W.B. (1984). Development and control of annealing textures in low-carbon steels. Int. Mater. Rev..

[5] Zheng X.F., Liao L.H., Kang Y.L., Liu W., Qiu Q.Q. (2018). The effect of chemical composition and processing technology on the microstructure, texture and earing behavior of DR tinplate. JMEPEG.

[6] Fang Y., Mo Z.Y., Sun C.F., Wu Z.G., Liu W., Song H. (2019). Effect of secondary cold reduction rate on microstructure and properties of tinplate. Iron Steel.

[7] Spišák E., Slota J., Kvaškaj T., Bobenič A. (2006). The influence of tandem mill reduction on double reduced (DR) tinplates anisotropy. Metalurgija-Sisak.

[8] Sun C.F., Fang Y., Wang Y.Q., Liu W., Pan H.W. (2019). Effect of coiling temperature on precipitation of secondary phase in ultra-low carbon T-3 CA steel. Iron Steel.

[9] Hu H., Si X., Ma L. (2014). Effects of technological parameters on grain size of hot rolling hard tinplate. Hot Work. Technol..

[10] Sun Z.H., Zhang P., Chen W., Gong Z.Q. (2015). Influence of the cold rolling reduction rate on microstructures and mechanical properties of the continuous annealing MR steel. Steel Roll..

[11] Meng D., Wang C., Zhong H., Yu G., Chen Q. (2016). Effect of cold rolling reduction on recrystallization annealing microstructure and properties of ultralow carbon deep drawing Steel. Hot Work. Technol..

[12] Zheng J., Hu Z., Long D. (2016). Effect of Reduction Ratio in Secondary Cold-rolling on Mechanical Properties and Texture of DCR Strip Steel. Mater. Rep..

[13] Černík M., Gburík R., Hrabčáková L., Vranec P. (2015). Texture analysis of tinplate steel and its application in production of double reduced high strength tinplate grades with controlled earing properties. IOP Conf. Ser. Mater. Sci. Eng..

[14] Alworth H.M., Michalak J.T., Shei S.A. (1987). The effects of second cold reduction on the plastic anisotropy, crystallographic texture and earing behavior of DR-9 Tin-Mill product. J. Appl. Metalwork..

[15] Hu X., Xia J., Zhou L., Wang L., Fang F. (2018). Effect of cementite morphology on pearlitic wire drawing deformation. J. Iron Steel Res..

[16] Ji P., Zhou L., Zhou X., Fang F., Jiang J. (2018). Study on anisotropic mechanical properties of cold drawn pearlitic steel wire. ACTA Metall. Sin..

[17] Abe H., Suzuki T., Takagi K. (1981). Effects of the size and morphology of cementite particles on the annealing texture in low-carbon aluminum-killed steel. Trans. Iron Steel Inst. Jpn..

[18] NIST X-ray Photoelectron Spectroscopy Database. http://srdata.nist.gov/xps.

[19] Zheng X., Yan Y., Kang Y. (2019). Comparative study of earing behavior in cold rolled-batch annealed and cold rolled-continuous annealed of double reduced (DR) tinplate steels. J. Harbin Inst. Technol..

[20] Liao L., Zheng X., Kang Y., Liu W., Yan Y., Mo Z. (2018). Crystallographic texture and earing behavior analysis for different second cold reductions of double-reduction tinplate. Int. J. Miner. Metall. Mater..

[21] Li S., Zhang X. (1996). A new method for predicting earing tendency of texture sheets. Acta Met. Sin..

[22] Inagaki H. (1991). Basic earing behaviour of fcc and bcc metals in cup drawing. Z. Fuer Met. Mater. Res. Adv. Tech..

[23] Xiang Z., Seki F., Ito K., Yoshinada N. (1995). Influence of dissolved carbonic content for (111) fiber texture in high purity and ultra-low carbonic 08Al deep drawing steel sheet. Iron Steel.

[24] Toge T., Muraki M., Komatsubara M., Obara T. (1998). Origin of recrystallization texture evolution and active slip systems in cold rolled 3% Si–Fe (100)[011] single crystal. ISIJ Int..

[25] Yan Y., Kang Y., Mo Z., Zheng X., Shen Y. (2017). Correlation between texture, earing behaviour and dissolved carbon and nitrogen. Mater. Sci. Technol..

